# A Case, Shewing the Efficacy of Flowers of Zinc, in the Epilepsy

**Published:** 1786

**Authors:** John Lind

**Affiliations:** Fellow of the Royal College of Physicians at Edinburgh, and Physician to the Royal Hospital at Haslar


X. *A Caſe, ſhewing the Efficacy of Flowers of Zinc, in the Epilepſy. By* John Lind, *M. D. Fellow of the Royal College of Phyſicians at Edinburgh, and Phyſician to the Royal Hoſpital at Haſlar. Communicated in a Letter to* William Wright, *M. D. F. R. S. and by him to Dr.* Simmons.

ON the third of May, 1779, Lieutenant S—p—d, was admitted into ſick quarters at Goſport for an epilepſy, under which he had laboured three years. It had proceeded from a fright. On its firſt attack, the fits had been irregular, returning generally once in three weeks or a month; of late they had obſerved an exact period, returning once a week, every Saturday. There was always a warning for twenty-four hours before the attack, by an increaſed ſenſibility of the ſyſtem and frequent convulſive twitchings. He was nineteen years of age, and had ſuffered much during the fits; but in the intervals enjoyed good health.

Bleed-

Bleeding, Bliſters, Bark, Valerian, Miſſeltoe, Cinnabar, and ſeveral medicines, with which he was unacquainted, had been tried without effect. On being admitted into ſick quarters a purge was given, and afterwards, night and morning, five grains of the flowers of zinc, made into a pill with conſerve of hips. By degrees the quantity of zinc was increaſed to ten grains, which was as much as the ſtomach would bear. Under this courſe, the fit which came on at the end of the firſt week was milder than uſual. Next week, inſtead of a true fit, he had only an increaſed ſenſibility of the nervous ſyſtem, and frequent convulſive twitchings, the uſual forerunner of a fit; and theſe did not begin as formerly on the day before the fit, but were confined to the day on which the fit, if not ſtopt by medicine, would regularly have happened. In the third week, at the expected return of the fit, the ſymptoms which marked its approach were leſs conſiderable than at the ſecond. In the fourth week they were very ſlightly felt. Upon perceiving them he always took a doſe of the zinc pills, which conſtantly removed every appearance. Sometimes he found one pill ſufficient for this, at other times two; if the complaints returned, he repeated

 the the pills a fecond or a third time, until every fymptom of uneafinefs was gone. He continued the ufe of the zinc pills twice a day for fix weeks, and had not any return of the fits, nor of the uneafy fenfation which threatened them; the days on which the fits would in their former courfe have returned paffing comfortably as the reft. At the expiration of the fix weeks he was difcharged perfectly well.

